# Cryptic intermediate snail host of the liver fluke *Fasciola hepatica* in Africa

**DOI:** 10.1186/s13071-019-3825-9

**Published:** 2019-12-04

**Authors:** Anna Mahulu, Catharina Clewing, Björn Stelbrink, Fred D. Chibwana, Immaculate Tumwebaze, J. Russell Stothard, Christian Albrecht

**Affiliations:** 1 Department of Animal Ecology and Systematics, Justus Liebig University Giessen, Giessen, Germany; 2 Department of Environmental Sciences, Zoological Institute, University of Basel, Basel, Switzerland; 3 Department of Zoology, University of Dar es Salaam, Dar es Salaam, Tanzania; 4 Department of Tropical Disease Biology, Liverpool School of Tropical Medicine, Liverpool, UK; 5 Department of Biology, Mbarara University of Science and Technology, Mbarara, Uganda

**Keywords:** Fascioliasis, Medical malacology, Cryptic species, *Galba truncatula*, Lymnaeidae, Dispersal, Islands-in-the-sky

## Abstract

**Background:**

Snails such as *Galba truncatula* are hosts for trematode flukes causing fascioliasis, a zoonosis that is a major public health problem. *Galba truncatula* has recently been shown to be a cryptic species complex. African populations of *Galba* spp. are not yet studied using molecular assessments and is imperative to do so and reconstruct the centre of origin of *Galba* and to understand when and by what means it may have colonized the highlands of Africa and to what extent humans might have been involved in that process.

**Methods:**

Samples from all known sub-ranges throughout Africa and new samples from Europe and Asia were obtained. We used a combination of two mitochondrial (*cox*1 and *16S*) and one nuclear (ITS2) markers and phylogenetic, divergence time estimates and phylogeographical methods to determine the identity and biogeographical affinities. We also reconstructed the colonization history including the likely mode of dispersal and tested for the presence of cryptic *Galba* species in Africa.

**Results:**

*Galba truncatula* is restricted to the Palaearctic region of the continent, namely Morocco. All sub-Saharan populations proved to be a distinct species according to the phylogenetic analyses and genetic distance. We propose to use the existing name *Galba mweruensis* (Connolly, 1929) for this species which is morphologically indistinguishable from the other two species hitherto known to occur in northern Africa, i.e. *G. truncatula* and *G. schirazensis*. Sub-tropical Africa has been colonized only once in either the Pliocene and possibly Miocene. Diversification within *G. mweruensis* is dated to the Plio-Pleistocene and thus human-mediated dispersal can be ruled out for the initial colonization of the isolated mountain ranges. There are potentially even more cryptic species in high altitude areas of Africa as outlined by the distinctness of the population found at the top of Mt. Elgon, Uganda.

**Conclusions:**

From a novel genetic inspection of available African material, a hitherto neglected distinct species, *G. mweruensis*, now appears a major host of *F. hepatica* throughout sub-Saharan Africa. A closer examination of trematode parasites hosted by this species is needed in order to understand transmission patterns in highlands throughout eastern and southern Africa. We encourage future studies to inspect other high altitudes areas in Africa in light of parasites of either veterinary or medical importance.
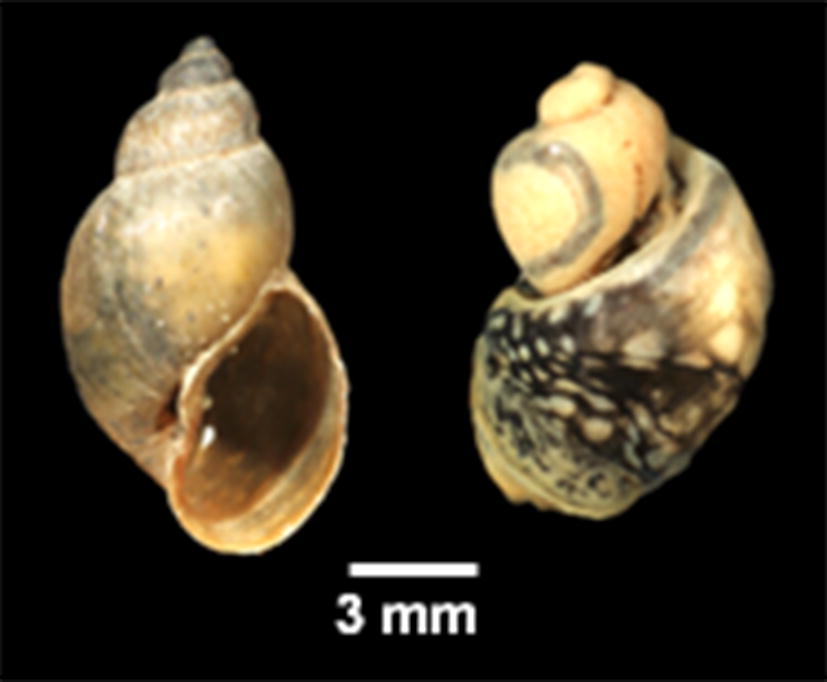

## Background

Parasitic disease caused by the liver flukes of the genus *Fasciola* affects hundreds of millions of people and livestock worldwide. Collectively, they cause considerable economic damage. Indeed, fascioliasis, a very debilitating snail-borne disease, is widespread across the globe; however, in the subtropical/cooler regions it is caused by *Fasciola hepatica* [1] whereas in the tropical/warmer regions is caused by *Fasciola gigantica* [2].

To complete the life-cycle, the two species of liver fluke are tied to a variety of intermediate freshwater pulmonate snail hosts of the family Lymnaeidae [3]. Until relatively recently, the taxonomy of snails was consolidated to a single genus *Lymnaea* with remarkable morphological diversity; however, with application of molecular phylogenetics a multi-generic nomenclature has become favoured with *Galba* and *Radix* now used in preference [4]. In Africa, for example, *Galba truncatula* (also known as *Lymnaea truncatula*) is involved in the transmission of *F. hepatica* while *Radix natalensis* is involved in the transmission of *F. gigantica* with any epidemiological cross-over considered to be rare [4]. As an intermediate host of *F. hepatica*, the liver fluke largely responsible for human disease, *G. truncatula* is characterized by its amphibious lifestyle, adaptation to cooler habitats, and its ability to withstand drought events and other harsh environmental conditions in unstable waterbodies [5]. It has been found in high altitudes in South America, where it can reach up to 4100 m [6] and it is thus among the few gastropods reaching extreme habitats on high elevations [7].

The taxonomy of lymnaeid gastropods continues to be debated [4, 8], but recent molecular phylogenetic studies improved the understanding of the evolution of this major freshwater gastropod family [3, 9–11]. The species *G. truncatula* has been treated as belonging to *Lymnaea* and *Fossaria* in North America and is thus a prime example of taxonomic confusion in lymnaeid systematics. *Galba truncatula* as the type-species of the genus is conceived to be mainly a Holarctic species [12], with a wide distribution range throughout North America and Eurasia, where it reaches as far as India [13]. The scattered occurrences in South America have been interpreted as recent introductions [14]. However, the real extent of the distribution of *G. truncatula* on a global scale is potentially masked by the occurrence of cryptic species that are morphologically indistinguishable from *G. truncatula*. Among these species are *Lymnaea cubensis* [15] and *Lymnaea schirazensis*, two species that have been previously confused with *G. truncatula* prior to the introduction of molecular methods of characterisation. Such a confusing situation has important implications to parasite transmission and epidemiology because the cryptic species may differ in their competence for transmission of *F. hepatica*.

Given the importance of these species for veterinary and human parasitology, a number of attempts have been made to identify species based on molecular markers. As a result, a relatively rich record of sequences of several mitochondrial and nuclear molecular markers is available for comparative analyses of material studied recently [3]. On the population level, SNPs [16] and microsatellites have been published [17]. A recent study proposed an easy and inexpensive PCR-based approach to distinguish among three cryptic *Galba* species [15].

Despite the variety of applicable molecular diagnostic markers, there is a significant gap of knowledge about snails referred to as *G. truncatula* on the African continent. Here, the *Galba truncatula*-like snails have a disjunct distribution with four largely isolated sub-ranges: in the mountainous parts of the Maghreb states in northern Africa [18], the highlands of Ethiopia [19], some highland areas in East Africa such as Mt. Elgon [20], Usambara Mt. [21], the Kitulo Plateau [22], the highlands of Lesotho [23], and temperate coastal, i.e. cooler, regions of South Africa [24].

**Fig. 1:**
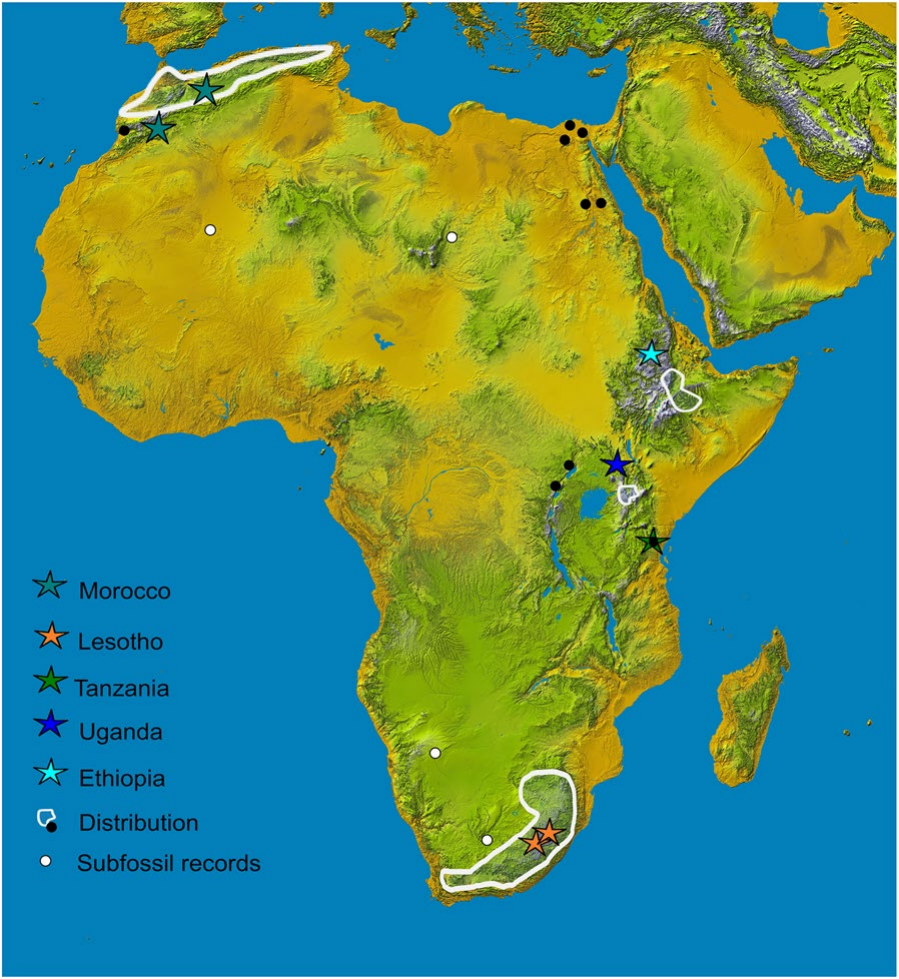
Distribution map of *Galba* in Africa including the sampling for the present study (see Table 1 for details). Four sub-ranges hitherto known of *Galba truncatula* are indicated (adopted from [21] and modified from [24, 26]. Note that occurrences on the Arab Peninsula are not shown here. Black dots denote isolated occurrences; white dots represent subfossil records. Localities of the newly obtained material are shown as coloured stars

When compared to the other native lymnaeid species in Africa, such as *Radix natalensis* the main host of *Fasciola gigantica*, the distribution pattern of what is considered *G. truncatula* is particularly striking (Fig. 1) being confined to allopatry in higher altitudes [20]. The discontinuous range of *G. truncatula* has been hypothesized to be the result of passive dispersal by migratory birds, being more likely perhaps than an alternative of much longer historical associations with geological vicariance of uplifted African high highlands [25]. Given scattered subfossil records in the Sahara, the Near East and Namibia [21], this could represent a range of ancestral or relic habitats isolated for eons. Another possibility would be a human- or livestock-mediated introduction, given the well-recognized anthropophily of the species [26]. In fact, historical records in the eastern part of the DR Congo have been attributed to human introductions [13]. Records of the Nile Delta in Egypt recently turned out to represent populations of *Lymnaea schirazensis* [27] and thus raise questions as to a potential camouflaged invasion in other parts of the continent. The only populations of *Galba* spp. that were identified by molecular DNA to be *G. truncatula* inhabited Mt. Elgon [20] and the Kitulo Plateau in southern Tanzania [22]. Both studies, however, used short fragments of the highly conservative nuclear ribosomal *18S* gene. Whereas, this genetic marker is sufficient to delimit *Galba* spp. from *Radix natalensis*, it is not suitable for intra-generic studies. Given this situation, it remains currently unclear whether the high-altitude African populations of *Galba* spp. indeed represent *Galba truncatula*. Moreover, it is unknown how these populations are related to populations in Europe, Asia and the Americas. Due to the complete absence of molecular assessments (but see [22]) it is, to date, impossible to reconstruct the centre of origin of *Galba* spp. and to understand when and by what means *Galba* spp. may have colonized Africa and to what extent humans might have been involved in that process.

To shed new light on the phylogeography of *Galba* spp. populations, and its impact on snail-borne diseases, we examine several African populations using combination of mitochondrial and nuclear DNA markers to determine the identity and biogeographical affinities, reconstruct the colonization history including the likely mode of dispersal, and test for the presence of cryptic *Galba* species in Africa.

## Methods

### Sampling

The snail specimens studied were collected in Africa between 2010 and 2018. Field trips were conducted in the Atlas Mountains in Morocco, the highlands of Ethiopia, the Eastern Arc Mountains of Tanzania, Mt. Elgon in Uganda and the highlands of Lesotho in southern Africa (Table 1). In addition, material from outside Africa available in the collection of University of Giessen Systematics and Biodiversity (UGSB) was also used. This included material from the type-locality of *G. truncatula* in Thuringia, Germany. Snails were manually collected using a scoop net in stable pools, ponds, marshes, swamps and slow-running waters. Specimens were fixed in 80% ethanol prior to DNA extraction.

**Table 1:** Locality, voucher (UGSB no.), and GenBank accession information for the species studied. UGSB is the acronym of the University of Giessen Systematics and Biodiversity collection

Species	Locality	Latitude	Longitude	Altitude (masl)	Code	UGSB no.	GenBank ID		
							*cox*1	*16S*	ITS2
*Galba mweruensis*	Lesotho, Mantsonyane	29.51682°S	28.29032°E	2212	Gmw15772	23470	MN601402	MN602685	MN602657
					Gmw15773	23471	MN601403	MN602686	MN602658
					Gmw15775	23473	MN601405	MN602688	MN602660
					Gmw15776	23474	MN601406	MN602689	MN602661
	Tanzania, Lushoto	04.44859°S	38.17837°E	1639	Gmw25316	20983	MN601423	MN602698	MN602674
					Gmw25317	20984	MN601424	MN602699	MN602675
					Gmw25318	20985	MN601425	MN602700	MN602676
					Gmw25319	20986	MN601426	MN602701	
	Ethiopia, Adi Aba Musa, Lake Ashenge	12.58650°N	39.52100°E	2409	Gmw22773	17407	MN601410	MN602707	MN602665
	Uganda, Budadiri, Mt. Elgon, Jackson’s Pool	01.14951°N	34.51054°E	3939	Gmw19054	12151	MN601409	MN602706	MN602664
	Uganda, Mt. Elgon	01.14954°N	34.54736°E	3792	Gmw26767	22833			MN602677
					Gmw26769	22835			MN602678
					Gmw26770	22836			MN602679
					Gmw26771	22837			MN602680
					Gmw26772	22838			MN602681
*Galba truncatula*	Morocco, Marrakech-Safi	31.15573°N	07.86678°W	2100	Gtr25298	18267	MN601412	MN602690	MN602666
	Morocco, Timdighas	32.68417°N	05.33972°W	1982	Gtr25297	18265	MN601411		
	Morocco, Marlay youssef Dam	31.39272°N	07.15383°W	167	Gtr25304	20971	MN601415		
					Gtr25305	20972	MN601416		
	Germany, Thuringia, Ilm River	50.89112°N	11.24089°E	289	Gtr15785	23475	MN601407	MN602704	MN602662
					Gtr15786	23476	MN601408	MN602705	MN602663
	Greece, Rhodos Island, 7 springs dam lake, on mud	36.25464°N	28.11596°E	232	Gtr25308	20975	MN601419	MN602694	MN602670
					Gtr25306	20973	MN601417		
					Gtr25307	20974	MN601418	MN602693	MN602669
	Russia, Ilovlya, river near Ilovlya Town	49.31367°N	43.97659°E	43	Gtr25312	20979	MN601420	MN602695	MN602671
	Russia, Moscow Region, Oka River	na	na		Gtr25313	20980	MN601421	MN602696	MN602672
					Gtr25314	20981	MN601422	MN602697	MN602673
	Slovenia, Vrhnika, creek Obrh	45.69906°N	14.51176°E	376	Gtr25299	18543	MN601413	MN602691	MN602667
					Gtr25301	18860	MN601414	MN602692	MN602668
	Nepal, Karnali	29.26667°N	82.15933°E	2300	Gtr11234	23477	MN601399	MN602702	MN602654
					Gtr12653	23478		MN602703	MN602656
	Nepal, Bagmati	29.30000°N	82.36667°E	2700	Gtr11235	23479	MN601400	MN602684	MN602655
	Nepal, Bheri	29.10717°N	82.58867°E	2625	Gtr11237	23481	MN601401		
	France, Limoges				GB2			HQ283236	HQ283262
*Lymnaea schirazensis*	Iran, Gilan Province, Taleb-Abad River				GB1		JF272607	JF272605	
*Lymnaea humilis*	USA, New York				GB3			FN182195	FN182191
*Lymnaea cousini*	Venezuela, Mucubají				GB4			HQ283237	HQ283266
*Lymnaea cubensis*	USA, South Carolina				GB5			FN182204	
*Lymnaea diaphana*	Argentina, Lago Escondido				GB6			HQ283241	HQ283260
*Lymnaea* sp.	Colombia, Antioquia				GB7			HQ283235	HQ283263
*Lymnaea viatrix*	Argentina, Rio Negro				GB8			HQ283239	HQ283265
*Radix natalensis*	Kenya, Kisumu, Lake Victoria	00.12739°S	34.74232°E	1140	Rna15771	23483	MN601427	MN602708	MN602708
*Pseudosuccinea columella*	South Africa, Mpumalanga	24.84539°S	30.83879°E	1374	Pco15787	23484	MN601428	MN602709	MN602683

*Abbreviations*: na, not available; masl, meters above sea level

### DNA extraction, amplification and sequencing

In most cases, DNA was extracted from two *Galba* specimens per locality. DNA extraction from ethanol-preserved snails was performed following the CTAB protocol of [28]. The primers used to amplify a fragment of the *cox*1 gene with a target length of 658 bp were LCO1490 and HCO2198 [29]. Amplification of the *LSU* rRNA fragment (*16S*) with a target length of 500 bp was performed with primers 16Sar and 16Sbr [30]. For the nuclear internal transcribed spacer ITS2, primers LT1 and ITS2-RIXO were used [9, 31].

PCR conditions were as described in [32]. Bidirectional sequencing was performed on an ABI 3730 XL sequencer at LGC Genomics, Berlin, Germany. *Galba* spp. samples successfully sequenced comprised two specimens from Germany, three specimens from Greece, two specimens from Slovenia, five specimens from Russia, six specimens from Nepal, one specimen from Ethiopia, five specimens from Lesotho, nine specimens from Morocco, four specimens from Tanzania, and six specimens from Uganda (Table 1).

### Phylogenetic analyses

DNA sequences were edited using MEGA v.7.0 [33]. The resulting dataset was complemented with other *Galba* spp. and *Lymnaea* spp. sequences available on GenBank (Table 1). The final dataset comprised a total of 19 specimens. The *16S* partition was aligned using the online program MAFFT [34], whereas Prankster [35] was used to align the ITS2 partition. The final concatenated alignment was 1494 bp long (*16S*: 434 bp; *cox*1: 655 bp; ITS2: 405 bp). Two outgroups were used for rooting the tree, *Radix natalensis* and *Pseudosuccinea columella* (Table 1).

We used jModelTest v.2.1.4 [36] to identify the best-fit substitution model for running phylogenetic analyses based on Bayesian inference (BI) as implemented in MrBayes v.3.2.6 [37]. Based on the corrected Akaikeʼs information criterion (AICc), the best-fit models were: GTR+Γ for *16S*, GTR+I+Γ for *cox*1, and GTR+Γ for ITS2. We ran two independent Markov Chain Monte Carlo (MCMC) searches (each with four chains) for 1 million generations and sampled every 50th tree and applied a ‘burn-in’ of 50%. Convergence of the two independent runs was examined *a posteriori* in Tracer 1.5 [38]. Effective sample size (ESS) values of > 200 indicated adequate sampling of posteriors distributions. In addition, a maximum likelihood (ML) analysis was conducted using RAxML-HPC2 8.2.10 [39] on the CIPRES Science Gateway [40] by applying the GTR+Γ model to all partitions and using a stop rule for the bootstrap analysis as recommended.

### Estimation of divergence times

Because of the scanty fossil record of *Galba* spp. and lymnaeids in general [4] and given the absence of a specific substitution rate for Lymnaeidae or freshwater pulmonate gastropods in general, we adopted a very conservative approach of dating the molecular phylogeny. We used two substitution rates for *cox*1, i.e. 1%/myr and 2%/myr and estimated divergence times using BEAST v.1.8.4 [41]. Analyses were run for 20 million generations, sampling every 1000th tree. Convergence of runs was analyzed using Tracer v.1.5. Because convergence was not reached and ESS values were < 200, we applied the less complex HKY substitution model to the different partitions (i.e. *16S*: HKY+Γ; *cox*1: HKY+I+Γ; and ITS: HKY+Γ). The maximum clade credibility (MCC) tree was identified using TreeAnnotator v.1.8.4 (BEAST package) by applying a ‘burn-in’ of 50%.

### Phylogeographical analyses

Phylogeographical analyses were carried out for the subset of samples from sub-Saharan Africa. The datasets consisted of 11 sequences for *cox*1, 11 sequences for *16S*, and 16 sequences for ITS2 and were individually analyzed. Relationships between haplotypes were calculated using a statistical parsimony network analysis performed using the software tool TCS v.1.21 [42] with a connection limit of 95%. Uncorrected genetic p-distances were calculated in MEGA v.7.0 [33] for within and among major *cox*1 clades inferred from the phylogenetic analyses.

## Results

### Phylogenetic analyses and divergence time estimation

**Fig. 2:**
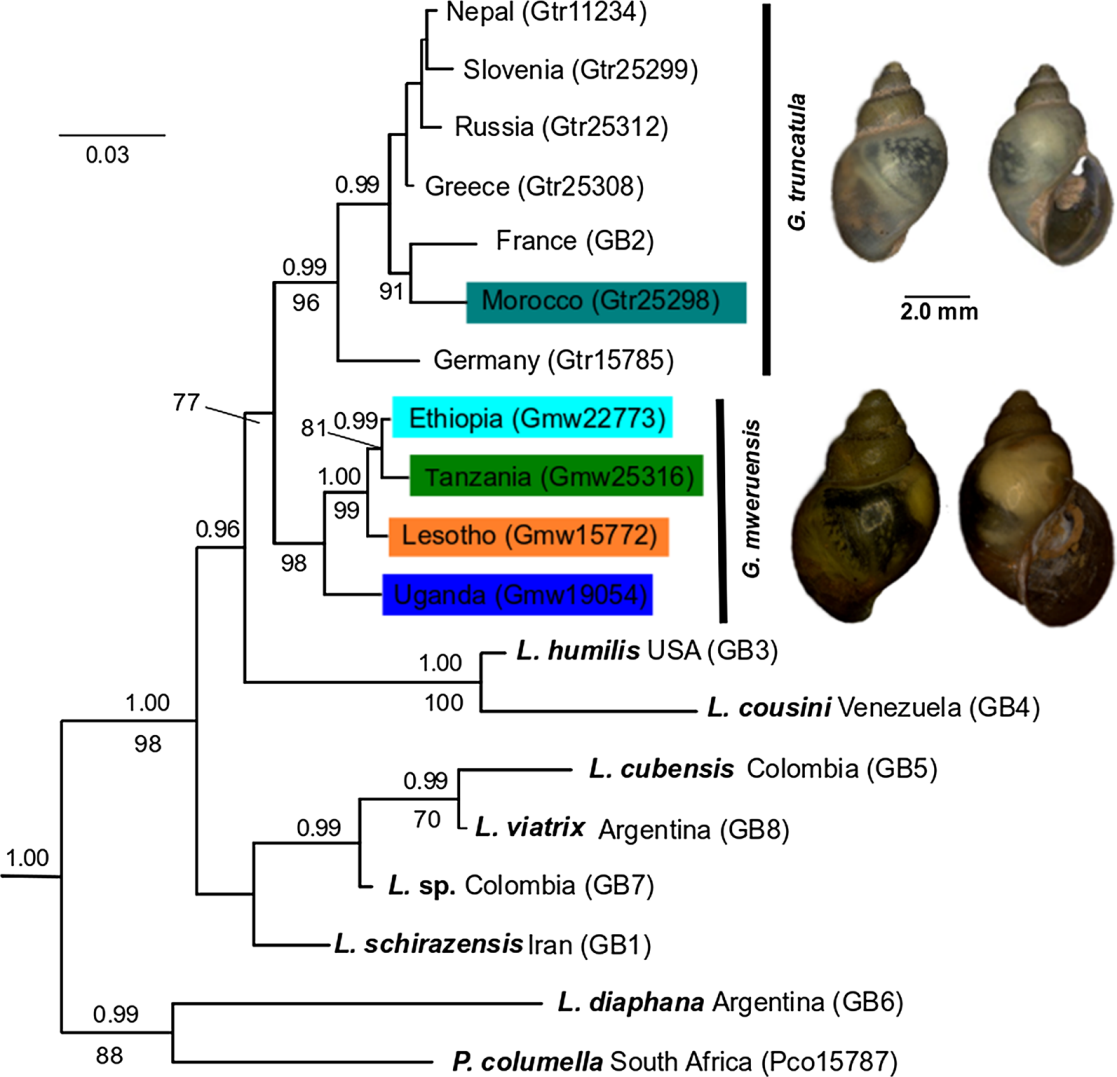
Bayesian inference phylogram based on concatenated *cox*1, *16S* and ITS2 sequences. The two outgroups have been removed *a posteriori*. Bayesian posterior probabilities are provided next to each node (top: MrBayes, bottom: RAxML). Sequences obtained from GenBank are labelled as GB1–GB8 (see Table 1). Nodes 1 and 2 indicate the nodes for which divergence time estimates are discussed. Colour codes used for species represent the origin of African samples and refer to those used in the map in Fig 1. Shell images are from *Galba truncatula* (Morocco, Gtr25298) and *Galba mweruensis* (Tanzania, Gmw25316). The scale-bar indicates substitutions per site according to the applied models of sequence evolution

The phylogenetic analyses conducted resulted in a generally highly supported phylogeny (Fig. 2) including a highly supported clade (ML bootstrap values, bs = 96; MrBayes posterior probability; pp = 1.00, BEAST posterior probability; bpp = 1.00) represented by *G. truncatula* comprising samples from Europe (including the type-locality in Thuringia, Germany), Asia, and a single specimen from Morocco. The remaining African samples formed a highly supported monophyletic clade (bs = 98; pp = 1.00; bpp = 1.00) that is referred to as *G. mweruensis* hereafter, which is possibly sister to *G. truncatula* (bs = 77, pp = 0.81, bpp = 1.00). *Galba mweruensis* (Connolly, 1929) is an available name for that clade ([43]; see Discussion). The distinction of *G. mweruensis* from *G. truncatula* is further corroborated by a more comprehensive *cox*1-based phylogeny (Additional file 1: Figure S1) and genetic distances (Table 2). However, both phylogenetic approaches (MrBayes and BEAST) revealed slightly different topologies. According to the MrBayes analysis, a clade of *Lymnaea humilis* and *L. cousini* was sister to the two *Galba* species. They together formed the sister-group to the remaining South American species (*L. cubensis*, *Lymnaea* sp., and *L. viatrix*). The cryptic species *G. schirazensis* from Iran and *L. diaphana* are more distantly related. In contrast, the BEAST analysis suggests a closer relationship of *G. schirazensis* (Iran) and *Lymnaea* sp. (Colombia) to *L. truncatula* and *L. mweruensis* and also found differences in the more basal phylogenetic relationships.

**Table 2:** Genetic distances of *Galba mweruensis* and *Galba truncatula* based on the *cox*1 dataset

	Uncorrected p-distance (%)			K2P model		
	*G. mweruensis*	*G. truncatula*	*G. mweruensis* vs *G*. *truncatula*	*G. mweruensis*	*G. truncatula*	*G. mweruensis* vs *G. truncatula*
Minimum	0.2	0.0	7.1	–^a^	–^a^	–^a^
Maximum	4.2	7.8	9.7	–^a^	–^a^	–^a^
Mean	2.3	3.2	8.4	2.4	3.4	9.0

*Note*: Uncorrected genetic p-distances and genetic distances based on the K2P model were calculated in MEGA v.7.0 [33]

^a^Not calculated

The split between *G. truncatula* and *G. mweruensis* was estimated to have occurred between *c*.3.9 (95% highest posterior density, 95% HPD: 5.6–10.2) and *c*.7.8 (95% HPD: 2.8–5.1) million years ago (Ma) depending on whether a clock rate of 2%/myr or 1%/myr was used (Additional file 2: Figure S2 and Additional file 3: Figure S3). The diversification of *G. mweruensis* started between *c*.1.7 (95% HPD: 1.1–2.3) and *c*.3.4 (95% HPD: 2.3–4.6) Ma.

### Phylogeographical analysis

**Fig. 3:**
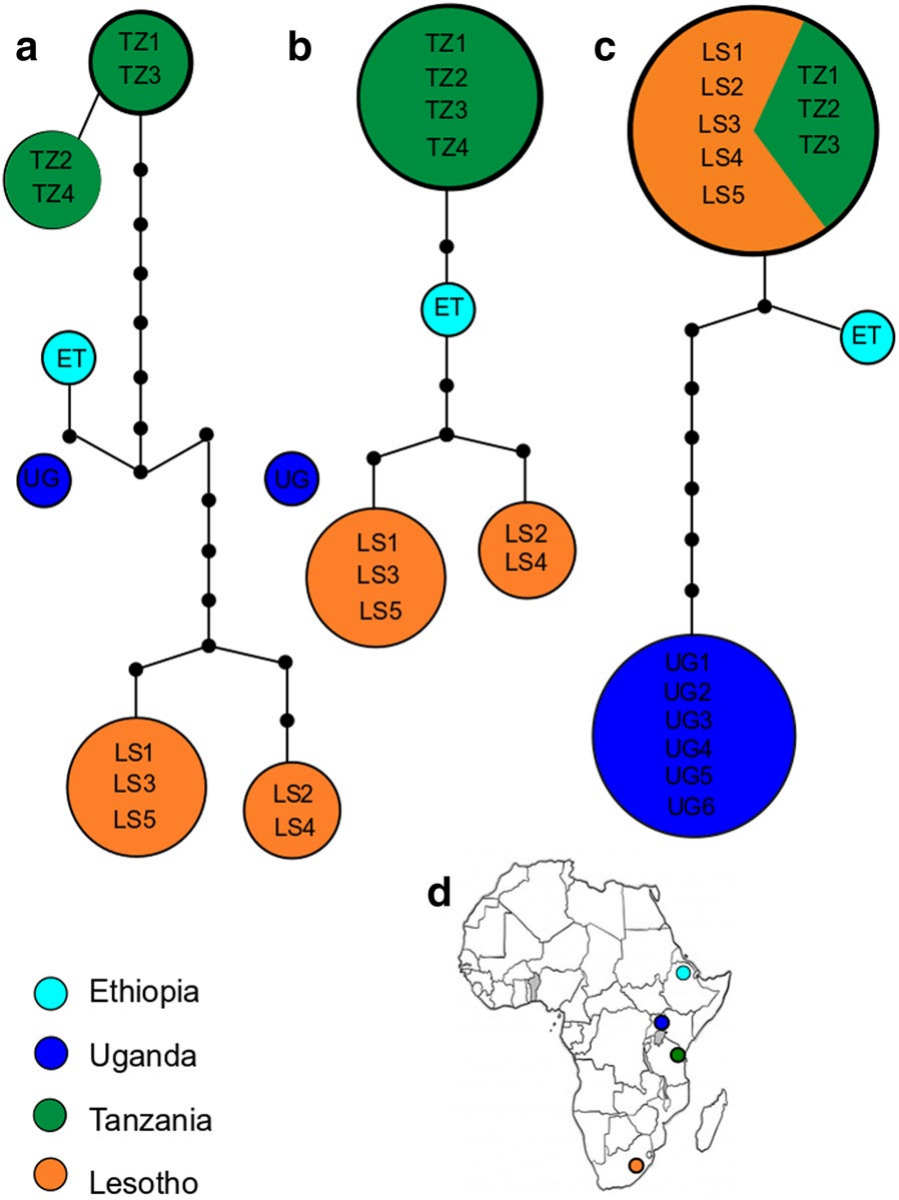
TCS maximum parsimony network of *Galba mweruensis* based on *cox*1 (**a**), *16S* (**b**) and ITS2 sequences (**c**). **d** Map showing the locations of the studied populations. The possible ancestral haplotypes are highlighted in bold, and the size of the circles corresponds to the number of individuals belonging to the respective haplotypes. Mutational steps representing missing haplotypes are displayed as small black circles

The *cox*1 haplotype network consisted of six haplotypes, two of which belonged to populations from Tanzania and Lesotho each, whereas the single specimens from Ethiopia and Uganda represented unique haplotypes. These geographical haplotypes were all connected except for the populations from Mt. Elgon (Uganda) that were separated by at least 22 mutational steps from the remaining haplotypes and thus represented a distinct haplotype network based on the 95% connection limit (Fig. 3). Similar patterns were also revealed by the *16S* and ITS2 datasets. Populations from Tanzania and Ethiopia seem to be more closely related in the two mitochondrial networks, whereas the ITS2 dataset suggested a closer relationship between populations from Ethiopia, Lesotho and Tanzania. The individuals from Mt. Elgon were also not connected with the remaining populations in the *16S* network (separated by at least 14 mutational steps) and were separated by 8 mutational steps from the other haplotypes in the ITS2 network based on the 95% connection limit.

The genetic distance within *G. truncatula* was higher (4.4%) than within *G. mweruensis* (1.9%). The uncorrected genetic p-distance between both groups was considerably high (9.0%).

## Discussion

### Identity of *Galba* in Africa and phylogenetic affinities

This study found two geographically separated species of *Galba* in Africa. *Galba truncatula* is restricted based on the available evidence to the Palaearctic zone of the continent, namely Morocco. All sub-Saharan populations proved to be a distinct species according to the phylogenetic analyses and genetic distance to the sister species *G. truncatula* from Europe and Asia. Interestingly, no *G. schirazensis* was found at the examined localities, which further supports the hypothesis that mountain ranges of tropical Africa are inhabited by a species different from *G. truncatula* and its cryptic counterpart *G. schirazensis* has not had opportunity to disperse into these areas or is unable to do so. We therefore propose to use the existing name *G. mweruensis* (Connolly, 1929) for this species that was described based on shell features and size measures (for a comparison of the original type-material and our new populations see Additional file 4: Figure S4; Additional file 5: Table S1). Moreover, it is morphologically indistinguishable from the other two species hitherto known to occur in Africa, i.e. *G. truncatula* and *G. schirazensis* (Additional file 6: Figure S5). *Galba mweruensis* is not the oldest available name for African *Galba* species for which even the section name *Afrogalba* had been introduced by Kruglov & Starobogatov [44]. Another taxon described earlier is *Galba umlaasianus* (Küster, 1862) from the Umlaas River, South Africa. Recent repeated attempts to obtain material from *terra typica* in the Kwa Zulu Natal Province of South Africa unfortunately failed. However, *G. umlaasiana* originally has been referred to as a lowland species of the temperate zones along the coastal regions of South Africa, whereas *G. mweruensis* has been described from mountainous terrain from Mweru town (type-locality) at the foothills of Mt. Kenya, which is somewhat in the core range of the species we found to occur widely in tropical Africa. Attempts to locate a population in the Mweru region in central Kenya in 2010 unfortunately failed. Moreover, Vinarski [45] compared both *G. mweruensis* and *G. umlaasiana* with the newly described *G. robusta* from Yemen and found the former two species to be morphologically different. We therefore propose to use the name *G. mweruensis* for mountainous *Galba* populations until it can be compared with topotypic material of *G. umlaasianus*. The latter taxon might even represent another distinct species given its different altitudinal range and may potentially co-occur with *R. natalensis* in the lower altitudes. Such a co-occurrence has not been observed for *G. mweruensis* in the studies that were conducted in the highlands of Lesotho (as *G. truncatula* in [24]), the Kitulo Plateau in Tanzania [22], and Mt. Elgon in Uganda [20]. In South Africa, however, either *G. truncatula* (*G. umlaasianus*), *L. natalensis* or the invasive *P. columella* have been reported to occur sympatrically [24].

Among the newly genotyped specimens of this species, the population from Mt. Elgon in Uganda is of particular interest. Mandahl-Barth [46] identified a small form of *Galba* at Mt. Elgon at 2770 m and attributed it to *G. mweruensis*. According to the present analyses, this population turned out to be sister to the remaining populations from Ethiopia, Lesotho and Tanzania, and the Mt. Elgon population was very distantly related to the remaining groups in the phylogeographical analyses. A more detailed analysis that investigates morphological and anatomical characters is needed in order to establish the status of the Mt. Elgon populations compared to their sub-Saharan counterparts. Hubendick [26] had material from the Kenyan slopes of Mt. Elgon and found similarities to *G. truncatula* but treated it as *G. mweruensis*. Isolated records of *Galba* spp. from the eastern part of the DR Congo west of Lake Albert and at Lake Kivu from considerably lower altitudes have not been confirmed during the last decades [21, 47].

The genetic diversity within *G. mweruensis* is comparable to that of other distinct *Galba* species such as *G. shirazenzis* [26]. Given the continuous and by far greater distributional range of *G. truncatula*, the higher degree of genetic differentiation in *G. truncatula* compared to *G. mweruensis* is not surprising. Nevertheless, the comparatively high genetic diversity within *G. mweruensis* raises the question as to how this diversity in isolated patches scattered over Africa has evolved and how these areas have been colonized. Further study in detail of several life-history traits for survival in cooler zones could be illuminating.

### Colonization history

Our study indicates that subtropical Africa has been colonized only once in either the Pliocene or even Miocene if one considers the age of the most recent common ancestor of *G. truncatula* and *G. mweruensis* as indicative of colonization time. Diversification within the African species *G. mweruensis* is dated to the Plio-Pleistocene and thus human-mediated dispersal can be ruled out for the initial colonization of the mountain ranges. We here applied commonly used substitution rates for mitochondrial markers in invertebrates, i.e. 1%/myr and 2%/myr (i.e. divergence rates of 2%/myr and 4%/myr). Assuming that *Galba* may have evolved with an extremely fast substitution rate of 4%/myr, the split would, of course, become younger (*c*.2 Ma). However, this would not change our conclusions that the hypothesis of human-mediated dispersal can be rejected. However, the data do not currently allow drawing a final conclusion as to whether Africa has been colonized from Europe, the Near East or South America. The tree topology may favour a colonization scenario out of Europe; however, Asian and especially Near East samples of *G. truncatula* are scarce and *G. robusta* (Yemen) could not be included. Subfossil records in Africa are also not very helpful as they originate from less mountainous regions and are not very informative given the small morphospace occupied by all *Galba* species. However, recent and subfossil Saharan records [18, 21] may indicate a stepping-stone dispersal for the northern Africa *G. truncatula* populations. The generally much higher lymnaeid diversity in the northern hemisphere makes an ‘out of Africa’ alternative for the *Galba* less likely. However, given the existence of the cryptic *G. schirazensis* in Egypt [27], no conclusion can be drawn here. On the intra-continental scale, a closer relationship between the Northeast and East African populations in comparison to the populations of the highlands of Lesotho would be expected. However, according to our analyses, specimens from Mt. Elgon are genetically more distinct compared to the remaining sub-Saharan haplotypes.

Dispersal by water birds, also at high altitudes, has been commonly shown to be a major factor in range evolution for freshwater molluscs in general [48] and pulmonate snails in particular [49]. To which extent water birds might have been involved in the colonization of these isolated mountain ranges can only be speculated. If such dispersal is as frequent as demonstrated in other regions [50, 51], *G. mweruensis* should be more widespread across different mountain ranges in sub-Saharan Africa.

Africa has experienced severe climatic fluctuations since the late Miocene and especially in the Plio-Pleistocene [52]. The patchy distribution pattern observed may thus reflect the emergence of climatic refugia in these mountain ranges that acted as islands in the sky [53]. Such relictary species distributions in African mountain ranges have been documented for diverse taxa such as birds [54], flightless insects [55] and frogs [56]. Although the status of *G. umlaasiana* has not been assessed yet, a correlation of cooler climates and the occurrence of *G. mweruensis* is apparent. Alternatively, the presence of the omnipresent and thus potentially competitive *R. natalensis* may considerably restrict the distribution of *G. mweruensis* to more temperate areas. Although mountain ranges are sometimes acting as refugia, they are also sensitive to climate changes [57]. Small and isolated populations might thus go through repeated bottlenecks and might experience local disappearance as found for the *Galba* population on Kitulo Plateau, Tanzania. A recent field survey (FC in October 2018) showed that the swampy habitats where the species earlier occurred [22] had completely dried out. A high estivating potential for *Galba* is, however, reported from highlands of Ethiopia [58].

### Parasitological implications of cryptic *Galba* species in Africa

Despite its patchy continental distribution, *G. mweruensis* is well established, especially in the extensive sub-ranges (Fig. 1). We here confirmed its presence in regions where it has not been observed for decades such as the Usambara Mountains (Tanzania) or Mt. Elgon in Uganda. It is also the predominant snail species in the highlands of Ethiopia and Lesotho and thus should be the intermediate host for livestock fascioliasis and potentially other trematode infections in that region [19, 59]. Dinnik & Dinnik [60] already pointed out that *G. mweruensis* is the intermediate host of both liver flukes, *F. hepatica* and *F. gigantica*, and thus not only represent major threats for livestock. For livestock, considerable economic losses are known from several African countries [61]. We suggest that there is a need to now ascertain the level of snail-parasite compatibility of *G. mweruensis* with several isolates of *F. hepatica* and *F. gigantica*, especially where these snails are found in cattle farmed areas.

Although estimating the prevalence of human fascioliasis is challenging [62], infection risks should be considered high wherever the intermediate host occurs [22]. Outbreaks can happen quickly, and the extent is often underestimated as recently outlined for the mountains in northern Tanzania [63]. Unlike with other human snail-borne diseases such as schistosomiasis, there is a high prevalence in high altitude regions. A prime example is the endemic in the Andean Altiplano [14, 64]. Although high mountainous regions are still considerably remote and less densely populated in Africa, there is a growing demand for land and thus humans increasingly occupying high elevations [65]. Even touristic activities such as trekking and mountain climbing are on the rise in basically all the mountain ranges where *G. mweruensis* occurs so further surveillance is warranted. Therefore, more dedicated surveys on infection and prevalence rates and the study of parasites actually hosted by *G. mweruensis* are necessary in all the areas where this species is established [20]. Whereas *G. schirazensis* is not particularly involved in transmission of *F. hepatica* [27], high rates of infection have been reported for *G. mweruensis* (originally *G. truncatula*) populations from Lesotho and Ethiopia [58, 66].

## Conclusions

This study has identified a hitherto neglected distinct species, *G. mweruensis*, as a host of *F. hepatica* throughout sub-Saharan Africa. It had previously been considered to be conspecific with Eurasian *G. truncatula*, a well-known and globally intermediate host species for several trematode parasites. Following our findings, a closer examination of the parasite communities hosted by *G. mweruensis* is needed in order to understand transmission patterns in highlands throughout eastern and southern Africa. Other high altitudes areas in Africa are to be surveyed for this species and veterinary and human health concerns have to be evaluated under the new precondition. It would be also interesting to study host specificity and potential climatic adaptations of both the host and the preferred temperature range of *F. hepatica* in Africa. The nature of striking non-overlap in occurrences between the omnipresent *R. natalensis* and *G. mweruensis* deserves more scientific attention because of its evolutionary implications and possible epidemiological cross-over as implicated host of *F. gigantica* and *F. hepatica*.

## Supplementary information

**Additional file 1: Figure S1.** Bayesian inference phylogram based on *cox*1. The two outgroups have been removed *a posteriori*. Bayesian posterior probabilities are provided next to each node (top: MrBayes, bottom: RAxML). Sequences obtained from GenBank are labelled plain whereas new sequences from this study are bold. Nodes 1 and 2 indicate the nodes for which divergence time estimates are discussed.

**Additional file 2: Figure S2.** BEAST molecular clock tree based on an HKY model and a substitution rate of 1%.

**Additional file 3: Figure S3.** BEAST molecular clock tree based on an HKY model and a substitution rate of 2%.

**Additional file 4: Figure S4.** Shell measurements of *Galba mweruensis* populations in comparison to the type specimen as described in Connolly, 1929 (p. 175).

**Additional file 5: Table S1.** Shell measurements of *Galba mweruensis* in the highlands of Lesotho, Tanzania and Mt. Elgon in Uganda.

**Additional file 6: Figure S5.** Shell, soft body anatomy and reproductive organs of *Galba mweruensis* from Lesotho (Mantsonyane). *Abbreviations*: BC, bursa copulatrix; PHT, phallotheca; PRP, praeputium; VD, vas deferens.

## Data Availability

All data generated or analysed during this study are included in the article and its additional files. The newly generated sequences were submitted to the NCBI GenBank database under the accession numbers MN601399–MN601428 for *cox*1, MN602684–MN602709 for *16S*, and MN602654–MN602683 for ITS2.

## Abbreviations

asl: above sea level
ESS: effective sample size
Gtr: *Galba truncatula*
Gmw: *Galba mweruensis*
Pco: *Pseudosuccinea columella*
PCR: polymerase chain reaction
Rna: *Radix natalensis*
UGSB: University of Giessen Systematics and Biodiversity collection
