# Supplementary figures and images for: Cryptic intermediate snail host of the liver fluke Fasciola hepatica in Africa
Source: Parasit Vectors. 2019 Dec 4;12:573. doi: 10.1186/s13071-019-3825-9 (PMC6894237; doi:10.1186/s13071-019-3825-9)

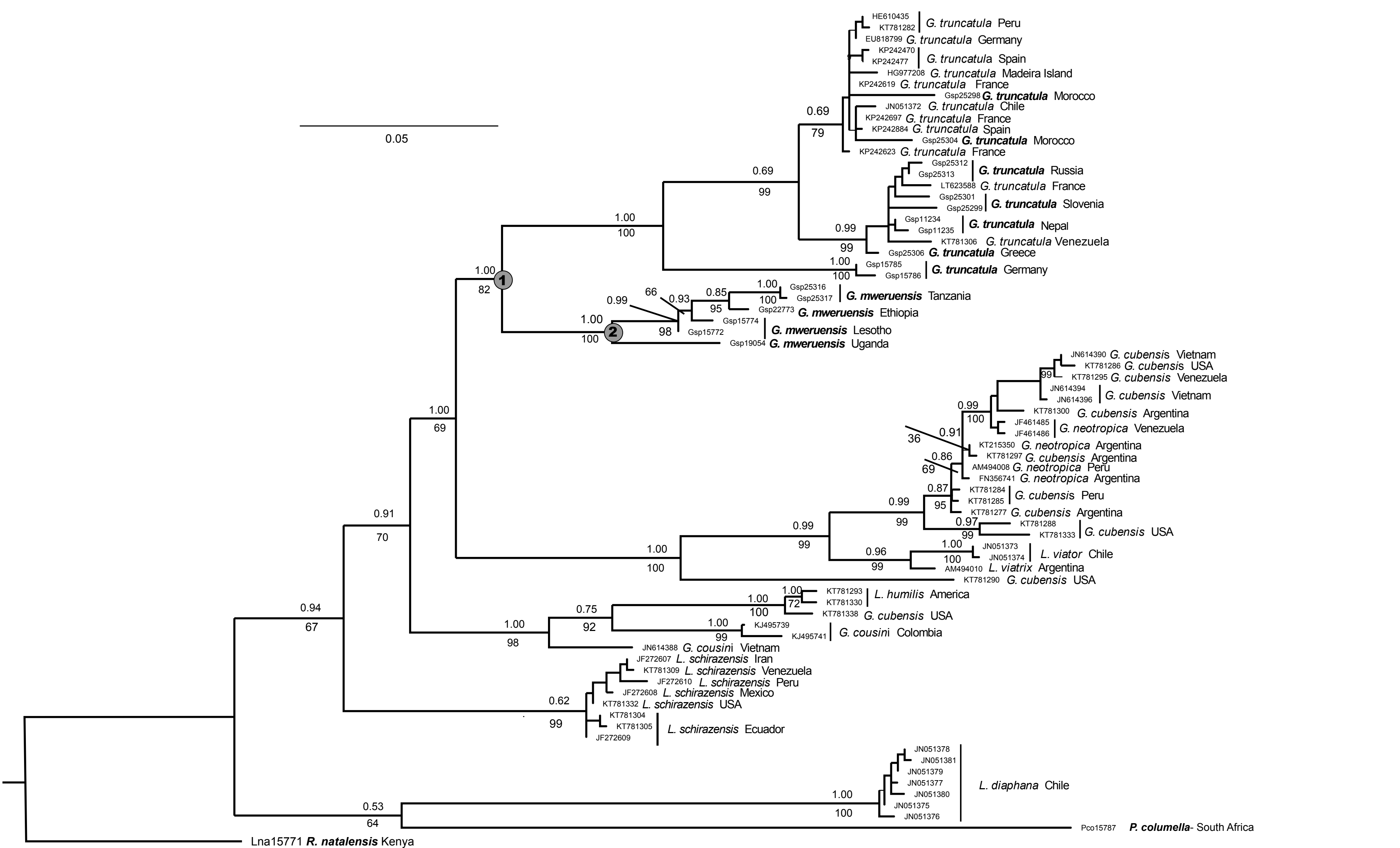

Supplement: Supplementary file 1 — Additional file 1: Figure S1. Bayesian inference phylogram based on cox1. The two outgroups have been removed a posteriori. Bayesian posterior probabilities are provided next to each node (top: MrBayes, bottom: RAxML). Sequences obtained from GenBank are labelled plain whereas new sequences from this study are bold. Nodes 1 and 2 indicate the nodes for which divergence time estimates are discussed. [file 13071_2019_3825_MOESM1_ESM.pdf]

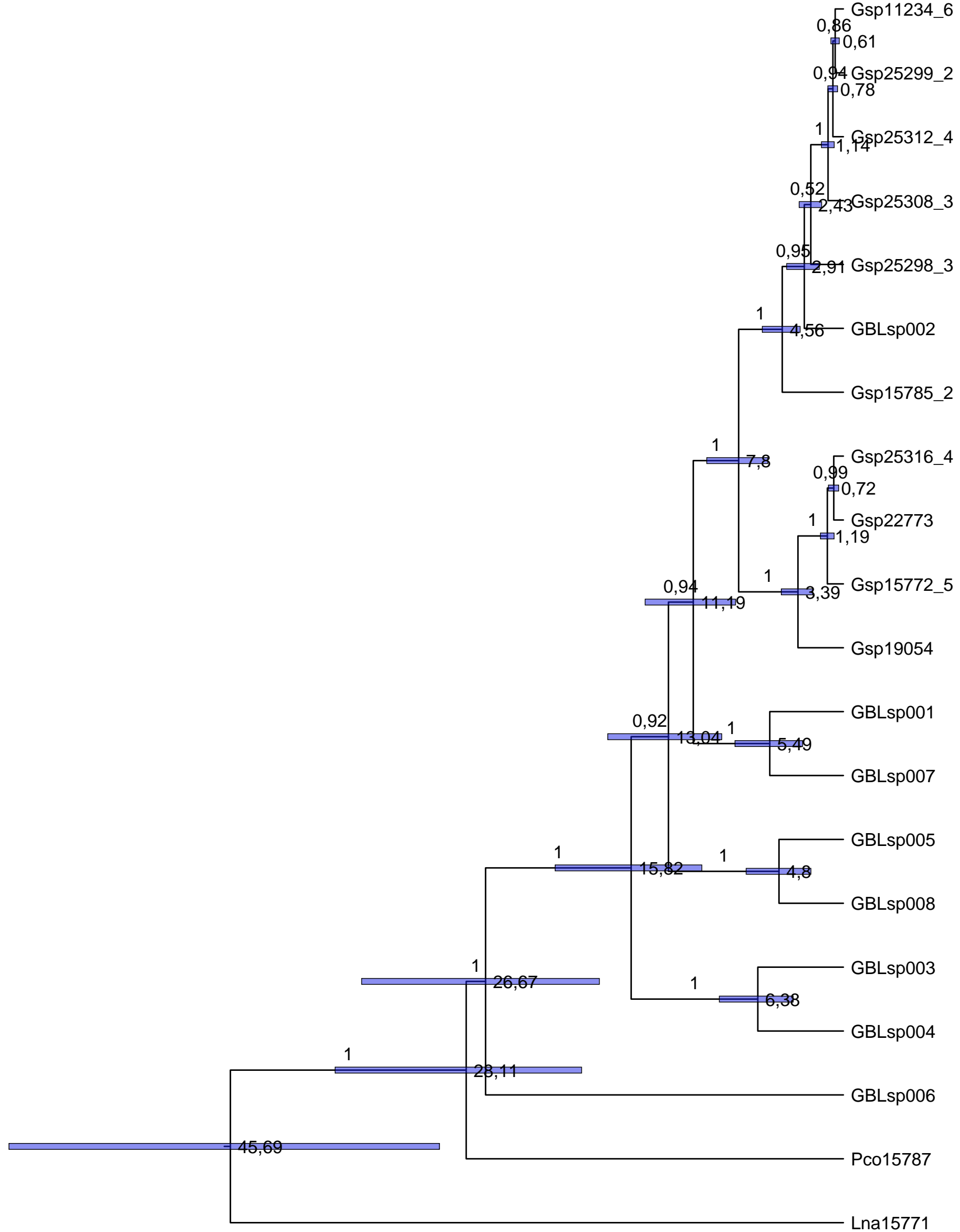

Supplement: Supplementary file 2 — Additional file 2: Figure S2. BEAST molecular clock tree based on an HKY model and a substitution rate of 1%. [file 13071_2019_3825_MOESM2_ESM.pdf]

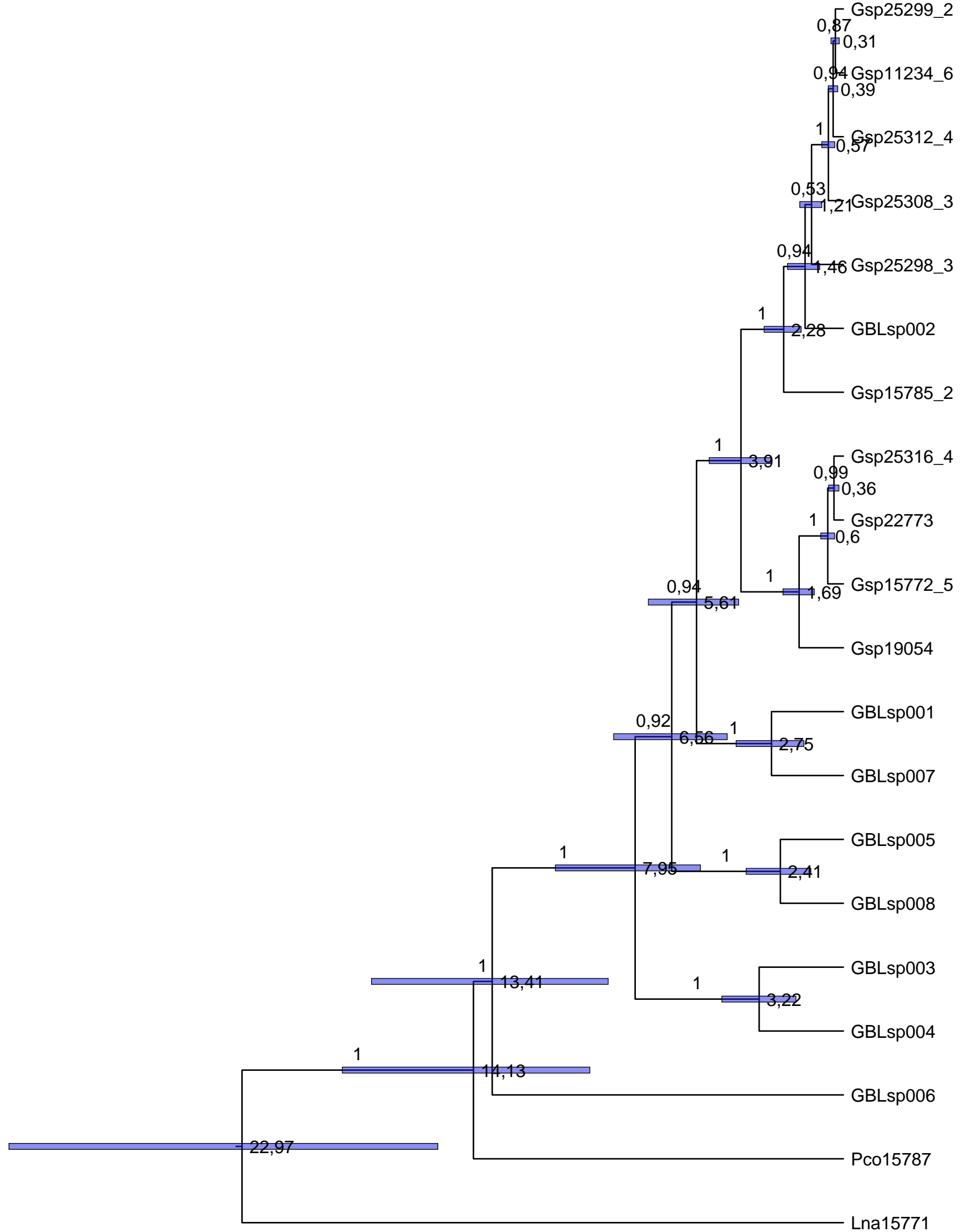

3.0

Supplement: Supplementary file 3 — Additional file 3: Figure S3. BEAST molecular clock tree based on an HKY model and a substitution rate of 2%. [file 13071_2019_3825_MOESM3_ESM.pdf]

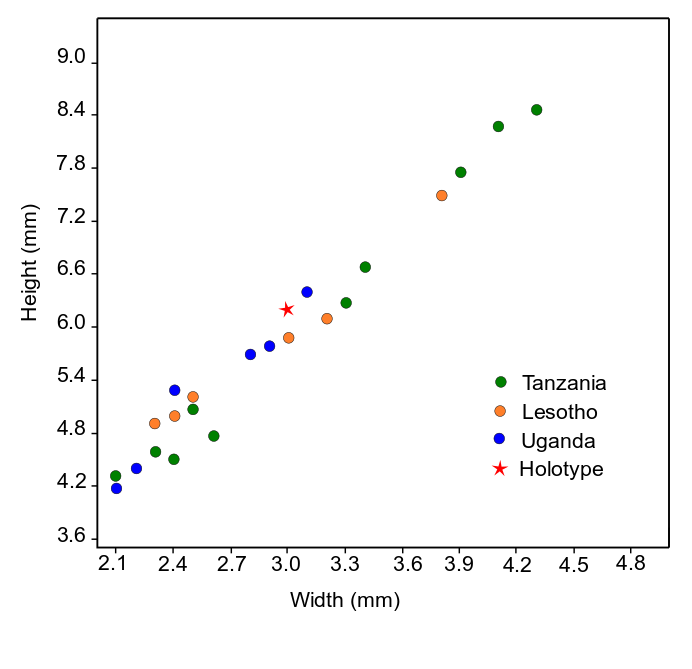

Supplement: Supplementary file 4 — Additional file 4: Figure S4. Shell measurements of Galba mweruensis populations in comparison to the type specimen as described in Connolly, 1929 (p. 175). [file 13071_2019_3825_MOESM4_ESM.png]

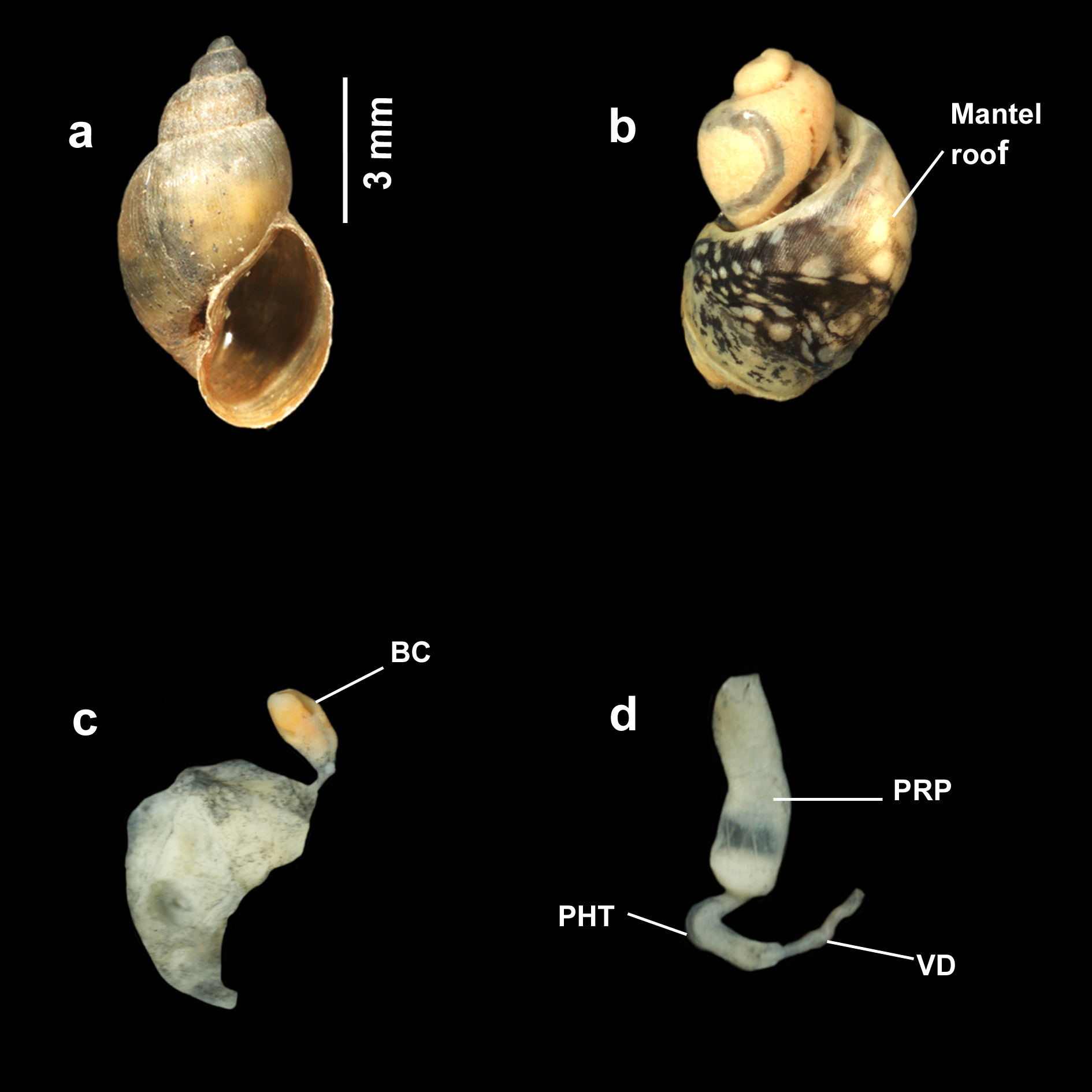

Supplement: Supplementary file 6 — Additional file 6: Figure S5. Shell, soft body anatomy and reproductive organs of Galba mweruensis from Lesotho (Mantsonyane). Abbreviations: BC, bursa copulatrix; PHT, phallotheca; PRP, praeputium; VD, vas deferens. [file 13071_2019_3825_MOESM6_ESM.png]
