# Supplementary material for: Cryptic intermediate snail host of the liver fluke Fasciola hepatica in Africa
Source: Parasit Vectors. 2019 Dec 4;12:573. doi: 10.1186/s13071-019-3825-9 (PMC6894237; doi:10.1186/s13071-019-3825-9)
Supplement: Supplementary file 5 — Additional file 5: Table S1. Shell measurements of Galba mweruensis in the highlands of Lesotho, Tanzania and Mt. Elgon in Uganda. [file 13071_2019_3825_MOESM5_ESM.docx]

**Additional file 5: Table S1.** Shell measurements of *Galba mweruensis* in the highlands of Lesotho, Tanzania and Mt Elgon in Uganda.

| Country | Specimens (N) | Mean height and s.d. (mm) | Mean width and s.d. |
| --- | --- | --- | --- |
| Lesotho | 8 | 5.8 (0.83) | 2.7 (0.57) |
| Tanzania | 10 | 6.1 (1.65) | 3.0 (0.81) |
| Uganda | 6 | 5.3 (0.85) | 2.6 (0.40) |
